# Effects of Corticosterone and Dietary Energy on Immune Function of Broiler Chickens

**DOI:** 10.1371/journal.pone.0119750

**Published:** 2015-03-24

**Authors:** Jiachang Yang, Lei Liu, Ardashir Sheikhahmadi, Yufeng Wang, Congcong Li, Hongchao Jiao, Hai Lin, Zhigang Song

**Affiliations:** 1 Department of Animal Science, Shandong Agricultural University, Taian, Shandong 271018, China; 2 Department of Animal Science, Faculty of Agriculture, University of Kurdistan, Sanandaj 66177-15175, Iran; 3 Laboratory of Livestock Physiology, Department of Biosystems, KU Leuven, Kasteelpark Arenberg, 30, 3001 Leuven, Belgium; University of New England, AUSTRALIA

## Abstract

An experiment was conducted to investigate the effects of dietary energy level on the performance and immune function of stressed broiler chickens (*Gallus gallus domesticus*). A total of 96 three-day-old male broiler chickens (Ross × Ross) were divided into two groups. One group received a high energy (HE) diet and the other group received a low energy (LE) diet for 7 days. At 5 days of age, the chickens from each group were further divided into two sub-groups and received one of the following two treatments for 3 days: (1) subcutaneous injection of corticosterone, twice per day (CORT group; 2 mg of CORT/kg BW in corn oil) and (2) subcutaneous injection of corn oil, twice per day (Control/Sham treatment group). At 10 days of age, samples of blood, duodenum, jejunum, and ileum were obtained. Compared with the other three groups, the LE group treated with CORT had the lowest average daily gain (ADG) and the poorest feed conversion ratio (FCR, *P* < 0.05). Furthermore, CORT treatment decreased the relative weight (RW) of the bursa independent of the dietary energy level, but it decreased the RW of the thymus only in the chickens fed the LE diet. By contrast, CORT administration decreased the RW of the spleen only in the chickens fed the HE diet (*P* < 0.05). The plasma total protein, albumin, tumor necrosis factor alpha, interleukin 2 and immunoglobulin G (IgG) levels were affected by the CORT treatment (*P* < 0.05); however, these factors were not significantly affected by the dietary energy level. Toll-like receptor-5 mRNA level was down-regulated by CORT injection in the duodenum and ileum (*P* < 0.05) and showed a trend of down-regulation in the jejunum (*P*=0.0846). The present study showed that CORT treatment induced immunosuppressive effects on the innate immune system of broiler chickens, which were ameliorated by consumption of higher dietary energy.

## Introduction

Stress is a widely used term in biology that describes a set of physiological and behavioral responses elicited by adverse stimuli. In intensive poultry production systems, broiler chickens face different types of stressors, such as high temperature, high stock density, and diseases that may impair productive performance and survival, thereby resulting in financial losses for farmers [1, 2].

Under stressful conditions, the total energy budget must be optimally divided between different physiological functions, such as thermoregulation, growth, and reproduction. In this context, the total energy balance or physiological condition may also determine the ability of individuals to mount an optimal immune response [3]. Immune functions have been traditionally regarded as maintenance requirements, but there is increasing evidence of the sensitivity of immunity to nutrient supply [4]. Moreover, the maintenance and deployment of the immune system may result in energetic or nutritional costs [5]. The experimental induction of the immune system increases energy expenditure and negatively affects energy metabolism [6].

The immune system is composed of two major subdivisions: the innate or non-specific immune system and the adaptive or specific immune system. The innate immune system constitutes the first line of defense to prevent invading microbes from entering host tissues, such as the gut [7]. In the gut, there is cross talk between microbiota and intestinal epithelial cells that relies on a large family of pattern recognition receptors (PRRs), including membrane-bound PRRs, for example dectin-1 and the Toll-like receptor (TLR) family. TLRs, such as the specific type I transmembrane receptors and pathogen pattern recognition receptors play very important roles in acute inflammation [8].

To the best of our knowledge, there is no report of the effects of stress on the mRNA expression of TLRs in the gastrointestinal tract of broiler chickens. Corticosterone (CORT) has been considered as the end product of HPA axis stimulation [9]. In poultry, CORT is the principal glucocorticoid (GC) involved in the regulation of fuel metabolism, feed intake, and immune responses [10]. Moreover, in broiler chickens, it has been reported that CORT treatment causes the marked regression of lymphoid tissues [11]. Thus, the present study was undertaken to assess the influence of the dietary energy density of a protein-adequate diet on the immune response of broiler chickens following CORT treatment.

## Materials and Methods

### Birds, housing, and experimental design

Male broiler chickens (Arbor Acres, *Gallus gallus domesticus*) were purchased from a local hatchery at 1 day of age and reared in an environmentally controlled room. The brooding temperature was maintained at 34°C (65% RH) for the first 2 days and then gradually decreased to 31°C (45% RH) until the chickens were 10 days of age. The light regime was 23L: 1D (light: dark). All chickens were fed the same commercial starter diet with 20.0% CP and 12.6 MJ ME/kg for the first 2 days [12]. All birds had free access to feed and water during the rearing period. This study was approved by the ethics committee of Shandong Agricultural University and conducted in accordance with the “Guidelines for Experimental Animals” of the Ministry of Science and Technology of China (Beijing, P. R. China).

At 3 days of age, 96 broiler chickens were evenly distributed over 16 floor pens with six birds per pen. The chickens in the 16 pens were either fed a high-energy (HE) or low-energy (LE) diet for 7 days. The compositions and nutrient levels of the experimental diets (on an air-dry basis) are listed in Table 1. At 7 days of age, the chickens from each group were randomly divided into two groups and received one of the following two treatments for 3 days: (1) subcutaneous injection of corticosterone, twice per day (2 mg CORT/kg BW in corn oil was injected each time; CORT group) and (2) subcutaneous injection of corn oil, twice per day (Control/Sham treatment group). The individual body weight and feed intake of each pen were recorded daily.

**Table 1:** Composition and nutrient levels of the experimental diets (on an air-dry basis).

Ingredients (%)	Low-energy diet	High-energy diet
Corn	46.62	44.86
Wheat bran	18.21	
Soybean oil		13.47
Soybean meal	30.68	36.98
NaCl	0.26	0.26
Mountain flour	1.89	1.74
CaHPO_4_	1.42	1.64
Choline chloride	0.26	0.26
Lysine	0.17	0.25
Methionine	0.24	0.29
Premix*	0.25	0.25
**Calculated nutrient content (%)**		
ME**	10.90	15.11
Crude protein	20.00	20.00
Crude fat	15.87	23.87
Calcium	1.00	1.00
Available phosphorus	0.45	0.45
Sodium chloride	0.25	0.25
Digestible lysine	0.96	1.07
Digestible methionine	0.53	0.56
Digestible methionine and cysteine	0.78	0.81
Digestible threonine	0.59	0.62

*Premix provides the following per kg of diet: VA, 8000 IU; VD_3_, 3000 IU; VE, 20 IU; VK, 2 mg; VB_1_, 4 mg; riboflavin, 8 mg; *D*-pantothenic acid, 11 mg; VB_5_, 40 mg; VB_6_, 4 mg; VB_12_, 0.02 mg; biotin, 0.15 mg; folic acid, 1.0 mg; choline, 700 mg; Fe (as ferrous sulfate), 80 mg; Zn (as zinc sulfate), 75 mg; Mn (as manganese sulfate), 80 mg; Cu (as copper sulfate) 10 mg, I (as potassium iodide), 0.40 mg; and Se (as sodium selenite), 0.30 mg.

** Metabolizable energy (MJ/Kg).

### Sampling and analysis

The production parameters of each group, including the average daily gain (ADG), average daily feed intake (ADFI), and feed conversion ratio (FCR), were calculated from 3 to 10 days of age. At 10 days of age, two chickens with similar body weights (193.00 ± 3.72 g) were selected from each pen. At 2 hours after injection, a blood sample was drawn from the wing vein using a heparinized syringe within 30 seconds and placed into an ice-cold tube (09:00 to 10:00). Plasma was collected as described previously [13] and stored at −20°C for analyses of total protein (TP), albumin (Alb), tumor necrosis factor-α (TNF-α), interleukin 2 (IL-2), and immunoglobulin G (IgG) levels. Immediately after obtaining the blood sample, the chickens were sacrificed by cervical dislocation [14], and the bursa, thymus, and spleen were harvested and weighed. Approximately 1 g to 2 g tissue samples were collected from the duodenum, jejunum, and ileum, snap-frozen in liquid nitrogen, and then stored at −80°C for RNA extraction.

Plasma concentrations of IgG (No. CH0101306), TP (No. CH0103001), and Alb (No. CH0103002) were spectrophotometrically measured using commercial diagnostic kits (Nanjing Jiancheng Biotechnology Institute, Nanjing, P. R. China). Plasma concentrations of TNF-α (No. GS-E60016) and IL-2 (No. GS-E60056) were measured using ELISA commercial diagnostic kits (Xinran Biology Technology Co., Ltd., Shanghai, P. R. China). For each plasma parameter, the samples from the four groups were evenly distributed in one plate in a single assay to eliminate inter-assay variability. The minimum detectable concentrations for the assays of IgG, TNF-α and IL-2 were 0.05 mg/L, 3 ng/L and 5 ng/L, respectively, and the intra-assay coefficients of variability were 9.5%, 13.4%, and 12.6%, respectively.

Gene expression (TLR5, TLR2–1, TLR2–2 and TLR4) was measured using real-time RT-PCR. Briefly, total RNA from the small intestine was extracted using Trizol (Invitrogen, San Diego, CA, USA). The quantity and quality of the isolated RNA were determined as described previously [15]. Reverse transcription was performed using a RT reaction mixture (10 μL) consisting of 500 ng of total RNA, 5 mmol/L MgCl_2_, 1 μL of RT buffer, 1 mmol/L dNTPs, 2.5 U AMV, 0.7 nmol/L oligo d(T), and 10 U ribonuclease inhibitor (TaKaRa Biotechnology, Co., Ltd. Dalian, P. R. China). cDNA was amplified using a 20-μL PCR reaction system containing 0.2 μmol/L of each specific primer (Sangon Biological Engineering Technology & Service Co., Ltd. Shanghai, P. R. China) and SYBR green master mix (TaKaRa Biotechnology, Co., Ltd. Dalian, P. R. China). Each cycle consisted of pre-denaturation at 95°C for 10 s, denaturation at 95°C for 5 s, and annealing and extension at 60°C for 34 s. Glyceraldehyde-3-phosphate dehydrogenase (GAPDH) was separately amplified as an internal control to normalize the differences in each sample. The primer sequences for chicken TLR5, TLR2–1, TLR2–2, TLR4 and GAPDH are listed in Table 2, and the primers were designed to span an intron to avoid genomic DNA contamination by Primer 5.

**Table 2:** Gene-specific primers used for analysis of chicken gene expression.

Gene^1^	GenBank No.	Primer sequences (5’→3’)	Product size (bp)	R value
TLR5	NM_001024586	F: TGTGGGAGAGAGGTTTATGTTTGG	169	0.997
		R: CTGAGAGAGAGGTGAGACAATAGG		
TLR2–1	NM_204278	F: ACGGTCATCTCAGCTACACCA	188	0.998
		R: GTCCAAACCCATGAAAGAGC		
TLR2–2	NM_001161650	F: CCAAGGAAGAGCTGACAGTG	243	0.995
		R: CAAAAGCGTCGTAGCAGATG		
TLR4	NM_001030693	F: CCACCGCGCTGACTCTTGGG	96	0.990
		R: TGGGGATGACCTCCAGGCACG		
GAPDH	NM_204305	F: CTACACACGGACACTTCAAG	244	0.997
		R: ACAAACATGGGGGCATCAG		

^1^Abbreviations: TLR5, toll-like receptor 5; TLR2–1, toll-like receptor 2–1; TLR2–2, toll-like receptor 2–2; TLR4, toll-like receptor 4; and GAPDH, glyceraldehyde 3-phosphate dehydrogenase.

The PCR products were verified by electrophoresis on a 0.8% agarose gel and DNA sequencing. Standard curves were generated using pooled cDNA from the assayed samples, and the comparative cycle threshold method (2^-ΔΔCT^) was used to quantify mRNA levels according to Livak and Schmittgen [16].

### Statistical analysis

The data are presented as the mean ± MSE. For body weight and feed intake analyses, n = 4/treatment; for plasma parameters, immune organ indices and gene expression, n = 8/treatment. A two-way ANOVA model was used to analyze the primary effects of CORT and the dietary energy treatments as well as their interactions, using Statistical Analysis Systems statistical software package (Version 8e, SAS Institute, Cary, NC, USA). When the primary effect of a treatment was significant, the differences between the means were assessed using Duncan’s multiple range analysis. The mean was considered significantly different at *P* < 0.05.

## Results

### Productive performance traits

As shown in Table 3, no effects of the interaction between CORT injection and the dietary metabolizable energy level on ADG, ADFI, and FCR were detected. However, the main effects of the dietary metabolizable energy level and CORT injection on ADG (*P* = 0.021, 0.033) and FCR were significant (*P* = 0.043, 0.012). ADFI did not differ between the four groups. CORT-exposed broiler chickens fed the LE diet had a lower ADG and a higher FCR compared with the other three groups (*P* < 0.05).

**Table 3:** Effects of corticosterone and dietary energy level on average daily gain, average daily feed intake and feed conversion ratio of broiler chickens from 3 to 10 days of age.

Item^1^	ADG (g)	ADFI (g)	FCR (g/g)
Energy level			
HE	23.11^a^	28.40	1.30^b^
LE	20.90^b^	29.15	1.41^a^
SEM	1.78	1.00	0.06
GC treatment			
None-CORT	23.15^a^	28.00	1.22^b^
CORT	20.90^b^	29.51	1.43^a^
SEM	1.82	1.01	0.05
Energy level × GC treatment			
HE-CORT	22.89	28.66	1.25
HE-none CORT	23.34	28.02	1.20
LE-CORT	18.89	30.28	1.60
LE-none CORT	22.90	27.94	1.22
SEM	3.66	2.03	0.12
Source of variance, *P>F*			
Energy level	0.021	0.278	0.043
GC treatment	0.033	0.396	0.012
Energy level × GC treatment	0.154	0.248	0.225

^1^Abbreviations: HE, high-energy diet; LE, low-energy diet; CORT, corticosterone; GC, glucocorticoid; ADG, average daily gain; ADFI, average daily feed intake; and FCR, feed conversion ratio.

^a^Means in a column and under each effect (main or interaction) with a common superscript do not differ significantly (P ≥ 0.05).

^b^Means in a column and under each effect (main or interaction) with a common superscript do not differ significantly (P ≥ 0.05).

### Plasma parameters

The levels of TP, Alb, TNF-α, IL-2, and IgG in the plasma were affected by the CORT treatment (*P* < 0.05); however, the dietary energy level did not affect these parameters (*P* > 0.05, Table 4). The CORT treatment enhanced the TP, IgG and Alb levels but reduced the TNF-α and IL-2 and levels. The interaction between CORT injection and dietary energy level was significant (P = 0.049) for the plasma IgG concentration. IgG levels in CORT-HE chickens were higher than in the other treatments.

**Table 4:** Effects of corticosterone and dietary energy level on immune-related factors in the plasma of broiler chickens.

Item^1^	TP (g/L)	Alb (g/L)	TNF-α (ng/L)	IL-2 (ng/L)	IgG (μg/mL)
Energy level					
HE	31.95	15.75	93.60	168.50	1231.45
LE	32.00	15.85	88.00	169.85	1057.65
SEM	3.50	1.10	5.65	13.01	141.32
GC treatment					
None-CORT	27.83^b^	14.42^b^	96.15^a^	179.85^a^	1044.20^b^
CORT	36.35^a^	17.00^a^	85.60^b^	158.35^b^	1245.09^a^
SEM	4.01	1.26	5.90	12.92	115.36
Energy level × GC treatment					
HE-CORT	35.86	17.23	89.84	148.26	1425.00^a^
HE-none CORT	27.85	13.96	97.11	188.25	1037.50^b^
LE-CORT	36.47	16.74	80.93	168.04	1050.00^b^
LE-none CORT	27.41	14.49	94.83	171.06	1064.88^b^
SEM	7.53	2.34	11.66	26.02	256.47
Source of variance, *P>F*					
Energy level	0.952	0.980	0.188	0.895	0.065
GC treatment	0.001	0.001	0.027	0.036	0.034
Energy level × GC treatment	0.755	0.415	0.429	0.069	0.049

^1^Abbreviations: HE, high-energy diet; LE, low-energy diet; CORT, corticosterone; GC, glucocorticoid; TP, total protein; Alb, albumin; TNF-α, tumor necrosis factor alpha; IL-2, interleukin 2; and IgG, immunoglobulin G.

^a^Means in a column and under each effect (main or interaction) with a common superscript do not differ significantly (P ≥ 0.05).

^b^Means in a column and under each effect (main or interaction) with a common superscript do not differ significantly (P ≥ 0.05).

### Immune organ relative weights

The CORT treatment decreased the relative weights of the bursa, thymus, and spleen (*P* < 0.01, Table 5). On the other hand, the dietary energy level had no significant effect on the relative weights of these organs (*P* > 0.05), and it did not interact with CORT for these factors.

**Table 5:** Effects of corticosterone and dietary energy level on the relative weights of immune organs of broiler chickens.

Item^1^	Bursa (%)	Thymus (%)	Spleen (%)
Energy level			
HE	0.19	0.35	0.74
LE	0.18	0.33	0.75
SEM	0.03	0.04	0.07
GC treatment			
None-CORT	0.23^a^	0.39^a^	0.86^a^
CORT	0.14^b^	0.28^b^	0.64^b^
SEM	0.02	0.04	0.08
Energy level × GC treatment			
HE-CORT	0.14	0.31	0.59
HE-none CORT	0.22	0.38	0.90
LE-CORT	0.13	0.25	0.68
LE-none CORT	0.24	0.39	0.82
SEM	0.05	0.08	0.016
Source of variance, *P>F*			
Energy level	0.772	0.370	0.999
GC treatment	0.001	0.001	0.001
Energy level × GC treatment	0.226	0.225	0.137

^1^Abbreviations: HE, high-energy diet; LE, low-energy diet; CORT, corticosterone; and GC, glucocorticoid.

^a-^Means in a column and under each effect (main or interaction) with a common superscript do not differ significantly (P ≥ 0.05).

^b^Means in a column and under each effect (main or interaction) with a common superscript do not differ significantly (P ≥ 0.05).

### Intestinal gene expression

The dietary energy level had no significant effect on TLR5, TLR2–1, TLR-2–2, or TLR-4 gene expression in the duodenum, ileum or jejunum (*P* > 0.05; Tables 6–8). However, the TLR-5 mRNA level was down-regulated by CORT injection in the duodenum and ileum (*P* < 0.05; Tables 6 and 8), and its level in the jejunum showed an obvious trend of down-regulation (*P* = 0.084; Table 7). The CORT treatment decreased mRNA levels of ileal TLR 2–1 (*P* < 0.05; Table 8), jejunal and ileal TLR 2–2 (*P* < 0.05; Tables 7 and 8). A significant interaction between CORT and energy level was observed for ileal TLR5, TLR2–1 and TLR2–2 (*P* < 0.05), and a tendency for the interaction of CORT with dietary energy level was observed for jejunal TLR5 (*P* = 0.069).

**Table 6:** Effects of corticosterone and dietary energy level on mRNA levels of TLR5, TLR2–1, TLR2–2, and TLR4 in the duodenum of broiler chickens.

Item^1^	TLR5	TLR2–1	TLR2–2	TLR4
Energy level				
HE	0.76	1.25	1.10	1.11
LE	0.79	1.23	1.11	1.05
SEM	0.18	0.28	0.14	0.17
GC treatment				
None-CORT	0.93^a^	1.11	1.07	0.96
CORT	0.62^b^	1.37	1.14	1.19
SEM	0.17	0.23	0.15	0.16
Energy level × GC treatment				
HE-CORT	0.67	1.28	1.07	1.28
HE-none CORT	0.85	1.21	1.13	0.92
LE-CORT	0.57	1.46	1.20	1.08
LE-none CORT	1.00	1.00	1.00	1.00
SEM	0.34	0.52	0.28	0.34
Source of variance, *P>F*				
Energy level	0.933	0.918	0.745	0.673
GC treatment	0.032	0.186	0.868	0.123
Energy level × GC treatment	0.346	0.336	0.170	0.302

^1^Abbreviations: HE, high-energy diet; LE, low-energy diet; CORT, corticosterone; GC, glucocorticoid; and TLR, toll-like receptor.

^a^Means in a column and under each effect (main or interaction) with a common superscript do not differ significantly (P ≥ 0.05).

^b^Means in a column and under each effect (main or interaction) with a common superscript do not differ significantly (P ≥ 0.05).

**Table 7:** Effects of corticosterone and dietary energy level on mRNA levels of TLR5, TLR2–1, TLR2–2, and TLR4 in the jejunum of broiler chickens.

Item^1^	TLR5	TLR2–1	TLR2–2	TLR4
Energy level				
HE	0.79	0.73	0.58	1.10
LE	0.81	0.87	0.73	0.96
SEM	0.16	0.19	0.17	0.09
GC treatment				
None-CORT	0.88	0.87	0.85^a^	1.01
CORT	0.71	0.73	0.45^b^	1.05
SEM	0.11	0.23	0.17	0.10
Energy level × GC treatment				
HE-CORT	0.81	0.71	0.45	1.17
HE-none CORT	0.77	0.74	0.70	1.02
LE-CORT	0.61	0.74	0.45	0.92
LE-none CORT	1.00	1.00	1.00	1.00
SEM	0.26	0.41	0.35	0.20
Source of variance, *P>F*				
Energy level	0.747	0.537	0.312	0.105
GC treatment	0.084	0.758	0.006	0.783
Energy level × GC treatment	0.069	0.525	0.263	0.221

^1^Abbreviations: HE, high-energy diet; LE, low-energy diet; CORT, corticosterone; GC, glucocorticoid; and TLR, toll-like receptor.

^a^Means in a column and under each effect (main or interaction) with a common superscript do not differ significantly (P ≥ 0.05).

^b^Means in a column and under each effect (main or interaction) with a common superscript do not differ significantly (P ≥ 0.05).

**Table 8:** Effects of corticosterone and dietary energy level on mRNA levels of TLR5, TLR2–1, TLR2–2, and TLR4 in the ileum of broiler chickens.

Item^1^	TLR5	TLR2–1	TLR2–2	TLR4
Energy level				
HE	0.85	0.81	0.72	1.10
LE	0.84	0.76	0.79	0.97
SEM	0.15	0.16	0.10	0.14
GC treatment				
None-CORT	0.93^a^	0.92^a^	0.88^a^	1.03
CORT	0.76^b^	0.66^b^	0.63^b^	1.04
SEM	0.95	0.12	0.11	0.10
Energy level × GC treatment				
HE-CORT	0.85^a^ ^b^	0.79^a^ ^b^	0.69^b^	1.15
HE-none CORT	0.85^a^ ^b^	0.82^a^ ^b^	0.75^b^	1.06
LE-CORT	0.67^b^	0.52^b^	0.57^b^	0.93
LE-none CORT	1.00^a^	1.00^a^	1.00^a^	1.00
SEM	0.20	0.29	0.22	0.25
Source of variance, *P>F*				
Energy level	0.879	0.330	0.983	0.150
GC treatment	0.035	0.019	0.003	0.922
Energy level × GC treatment	0.035	0.020	0.021	0.416

^1^Abbreviations: HE, high-energy diet; LE, low-energy diet; CORT, corticosterone; GC, glucocorticoid; and TLR, toll-like receptor.

^a^Means in a column and under each effect (main or interaction) with a common superscript do not differ significantly (P ≥ 0.05).

^b^Means in a column and under each effect (main or interaction) with a common superscript do not differ significantly (P ≥ 0.05).

## Discussion

It is well known that decreased feed intake is a primary cause of reduced growth rate in broiler chickens. Previous studies have reported that stressful conditions induce reductions in growth rate even when feed intake levels are maintained [17, 18]. In the present study, exposing broiler chickens to CORT decreased the ADG in the absence of a change in feed intake. Physiological and biochemical changes may have been responsible for the lower growth rate of the CORT-exposed broiler chickens. Increased energy expenditure and protein oxidation have been suggested as possible reasons for the suppressive effects of GCs on the growth rate of these animals [19]. Furthermore, it has been reported that GCs may suppress growth by reducing the absorption of feed through the small intestine [20]. In the present study, feeding broiler chickens a high-energy diet did not compensate for the adverse effects of CORT-induced stress on ADG and FCR. Furthermore, the decrease in the growth rate in the absence of a reduction in feed intake of the CORT-exposed broiler chickens fed the LE diet resulted in a higher FCR.

Stressors can modify plasma levels of albumin and gamma globulins (predominantly IgG) [21]. Furthermore, previous studies have shown that GCs, as the end product of the activated HPA axis, increase albumin synthesis *in vivo* [22] and up-regulate albumin gene expression *in vitro* [23]. In this study, the CORT treatment increased the plasma albumin concentration in the CORT-exposed broiler chickens regardless of the dietary energy level. However, increasing the dietary energy level did not increase the plasma albumin level.

Although the effect of the dietary energy level on the plasma IgG level was not significant, the level of plasma IgG in the CORT-exposed broilers fed the high-energy diet (CORT-HE) significantly increased compared with the other three groups. An effect of the dietary energy level on the immune system of broiler chickens has been reported by Mirzaaghatabar et al. [24]. In this study, broiler chickens fed the HE diet had a higher plasma IgG concentration than those fed the LE diet. However, in the present study, to formulate the HE diet, soybean oil was used as a dense, high-energy ingredient. Soybean oil contains more than 50% linoleic acid, which is an n-6 polyunsaturated fatty acid (PUFA). Previous studies have shown that different PUFAs can modulate the immune system in poultry [25, 26]. Sijben et al. [27] have demonstrated that a low α-linolenic/high linoleic acid diet increases the antibody response against keyhole limpet hemocyanin in growing layer hens. Parmentier et al. [28] have shown that a sunflower oil-enriched diet increases the plasma IgG concentration against bovine serum albumin. Sunflower oil is a known source of linoleic acid. Hence, it should be taken into account that the increased plasma level of IgG in the broiler chickens fed the HE diet could have been due to a high level of linoleic acid in the soybean oil that was used to formulate this feed.

In addition to the effects of GCs on the growth rate and plasma proteins, in broiler chickens, the administration of GCs resulted in the rapid degeneration of lymphoid tissues, such as the thymus and bursa, due to their apoptotic effects [29, 30]. The mechanism behind glucocorticoid-induced apoptosis has not yet been fully elucidated. However, it may vary depending on the cell type and the expression levels of GC receptors (GRs) [31, 32]. Schaumburg and Crone [33] have demonstrated that bursa lymphocytes in chickens contain higher levels of GRs than those of the thymus (1200 sites/cell versus 600 sites/cell), which makes the bursa more susceptible to the effects of GCs. In this study, the CORT treatment reduced the bursa and thymus weights of the birds fed the LE diet. However, compared with the none-CORT-injected group, the reduction in the weight of bursa in the CORT-injected group was higher than the observed reduction in the thymus weight (39% for bursa vs 28% for thymus).

Additionally, GCs specifically inhibit glucose transport in a variety of peripheral tissues, such as skeletal muscle, adipocytes, and endothelial cells [34, 35]. It has been reported that bursal cells have a high priority for glucose, isoleucine and lysine, but thymic cells have a very low priority for these nutrients [36, 37]. Although we did not measure glucose transport to the lymphoid organs in the present study, it is likely that CORT inhibited glucose transport to lymphoid cells (both bursa and thymus cells); however because of the higher priority of bursa cells for glucose, this organ showed significant tissue regression and weight loss. In addition to the decreased relative weights of the bursa and thymus, in the present study, the relative weight of the spleen was reduced in the broiler chickens treated with CORT in comparison with their corn oil-exposed counterparts. In mice, it has been reported that endogenous and extrinsic GCs cause apoptosis in the spleen and that this may be one mechanism by which stress responses cause immunosuppression [38].

Activation of the HPA axis and the release of GCs alter immune function by down-regulating inflammatory cytokines and up-regulating anti-inflammatory cytokines [39, 40]. Dietary energy concentration and intake influence the immune function [41] of stressed animals. In the present study, the CORT treatment reduced the plasma IL-2 concentration. IL-2, which is a member of the pro-inflammatory cytokine family, is necessary for the growth, proliferation, and differentiation of T cells. In addition to plasma IL-2, CORT-exposed broiler chickens had lower plasma TNF-α level. TNF-α is a pro-inflammatory cytokine that is naturally produced by activated macrophages, monocytes, T cells, natural killer cells, and neutrophils.

Furthermore, it is known that TNF-α is a stimulator of nuclear factor kappa-β (NF-ĸβ), which is a key factor in the inflammatory response in a variety of cells [42]. In the intestine, NF-ĸβ activation is the end result of the TLR signaling pathway. Previous studies of mammals have shown that GCs alter TLRs in various peripheral cell types [43, 44]. In humans, dexamethasone, which is a synthetic GC, in combination with IFN-γ or TNF-α, synergistically enhance TLR2 expression in respiratory epithelial cells [45]. However, MacRedmond et al. [46] has shown that dexamethasone down-regulates TLR4 expression in an airway epithelial cell line. Limited information about the effects of GCs on TLRs in broiler chickens is available. In addition, the effects of GCs on intestinal TLRs have not yet been investigated. In this study, CORT treatment altered the mRNA levels of some intestinal TLRs. However, the gene expression pattern of TLRs is intestinal section-specific, which has also been observed in previous reports. In mice, TLR2 mRNA is predominantly expressed in the distal small intestine and proximal colon; however, TLR4 is predominantly expressed in the distal colon [47]. Bocker et al. [48] have reported that TLR4 mRNA is predominantly expressed in the colon, with its expression being weak and variable. The compartmental expression of TLR2 and TLR4 has not been reported in the small intestine of broiler chickens. Our data showed that TLR2–1, TLR2–2, TLR4, and TLR5 were expressed in all parts of the small intestine. However, no significant changes in the gene expression of TLRs in the duodenum of CORT-exposed broiler chickens were observed, except for a reduction in the mRNA level of TLR5 in the CORT-exposed broiler chickens. This observation can be a possible reason for the differences in the gene expression of TLRs in different parts of the gastrointestinal tract. Bacterial proliferation in the jejunum and ileum of broiler chickens is higher compared with the duodenum. Moreover, the sensing of pathogens by TLRs is more critical for broiler chickens to maintain intestinal homeostasis. Hence, the relatively higher expression of TLR4 in the ileum may increase mucosal protection against pathogens.

In this study, exposing broiler chickens to CORT significantly decreased the mRNA levels of ileal TLR 2–1, jejunal and ileal TLR2–2. TLR2 is an important TLR that controls mucosal inflammation by regulating the epithelial barrier function [49]. In addition, inhaled corticosteroids do not alter the mRNA level of TLR-2 in human lungs [50]. The functional properties of TLR2–2 in chickens are similar to those of mouse and human TLR2 [51]. The reduced jejunal and ileal TLR2–2 mRNAs in CORT-exposed broiler chickens implies that CORT treatment can affect the epithelial barrier functions of the jejunal and ileal mucosa, decrease the ability of TLR2 to respond to antigens, and impair the innate immune system in the intestinal mucosa. Moreover, the mRNA level of TLR5 decreased in the ileum of CORT-exposed broiler chickens fed the LE diets. No decrease in ileal TLR5 mRNA expression was observed in the broilers fed the HE diet after the CORT treatment in comparison with the birds fed the LE diet, which is likely due to the high level of n-6 fatty acids comprising the HE diet. TLR5 binds to bacterial flagellin and activates the pro-inflammatory response and secretion of proinflammatory cytokines [52]. Host recognition of flagellin has been reported to promote rapid neutrophil recruitment that protects the host from pathogens [53, 54].

Overall, the present findings showed that exposure to CORT induced the modulation of the innate immune system of broiler chickens, but it was ameliorated by higher dietary energy. However, due to the impacts of soybean oil (a rich source of linoleic acid) on plasma cytokines and the microbial population in the intestines, the high level of soybean oil in the broilers fed the HE diet should be taken into account. Additional studies are needed to acquire a better understanding of the mechanisms involved in the effects of GCs on the intestinal innate immune system of broiler chickens.
